# Aortic root surgery in septuagenarians: impact of different surgical techniques

**DOI:** 10.1186/1749-8090-4-17

**Published:** 2009-04-21

**Authors:** Nawid Khaladj, Rainer Leyh, Malakh Shrestha, Sven Peterss, Axel Haverich, Christian Hagl

**Affiliations:** 1 Department of Cardiac, Thoracic, Transplantation and Vascular Surgery, Hannover Medical School, Hannover, Germany

## Abstract

**Background:**

To evaluate the impact and safety of different surgical techniques for aortic root replacement (ARR) on early and late morbidity and mortality in septuagenarians undergoing ARR.

**Methods:**

Ninety-five patients (73.8 ± 3.2 years) were operated and divided into three groups according to the aortic root procedure; MECH-group (n = 51) patients with a mechanical composite graft, BIO-group (n = 22) patients with a customized biological composite graft, and REIMPL-group (n = 22) patients with a valve sparing aortic root reimplantation (David I). In 42.1% (40/95) of these patients the aortic arch was replaced. Follow-up was completed in 95.2% (79/83) of in-hospital survivors.

**Results:**

Hospital mortality was 12.6% (12/95) in the entire population (MECH. 15.7% (8/51), BIO 19.7% (4/22), REIMPL 0% (0/22); p = 0.004). Two patients died intraoperatively. The most frequent postoperative complications were prolonged mechanical ventilation ((>48 h) in 16.8% (16/93) (MECH. 7% (7/51), BIO 36.4% (8/22), REIMPL 4.5% (1/22); p = 0.013) and rethoracotomy for postoperative bleeding in 12.6% (12/95) (MECH. 12% (6/51), BIO 22.7% (5/22), REIMPL 4.5% (1/22); p = 0.19). Nineteen late deaths (22.9%) (19/83) (MECH 34.8% (15/43), BIO 16.7% (3/18), REIMPL 4.5% (1/22); p = 0.012) occurred during a mean follow-up of 41 ± 42 months (MECH 48 ± 48 months, BIO 25 ± 37 months, REIMPL 40 ± 28 months, p = 0.028). Postoperative NYHA class decreased significantly (p = 0.017) and performance status (p = 0.027) increased for the entire group compared to preoperative values.

**Conclusion:**

Our data indicate that valve sparing aortic root reimplantation is safe and effective in septuagenarians, and is associated with low early and late morbidity and mortality.

## Background

Aortic root replacement has become a generally accepted treatment modality for a variety of aortic root pathologies. Basically, two different surgical procedures and their modifications have been advocated. Composite graft valve replacement (CVR) of the aortic root applying different techniques for reimplantation of the coronary ostia, and different types of valve sparing aortic root replacement techniques [1-5]. Although results of these techniques have been extensively described only limited data from the literature are available about these surgical techniques in older patients [6-13]. Furthermore, different techniques for aortic root replacement in septuagenarians have not been evaluated yet. Thus, the aim of this study was to evaluate the impact and safety of different surgical techniques for aortic root replacement (ARR) on early and late morbidity and mortality as well as functional status in septuagenarians undergoing aortic root replacement.

## Patients and methods

This study was approved by the institutional review board; all patients gave written informed consent.

Between 1988 and 2003, 95 patients 70 years of age or older underwent aortic root replacement at our institution. Preoperative patient's characteristics are depicted in Table 1. According to the aortic root procedure the patients were divided into three groups. MECH-group (n = 51) patients who received a mechanical composite valve graft, BIO-group (n = 22) patients who received a customized biological composite valve graft, and REIMPL-group (n = 22) patients who received a valve sparing aortic root reimplantation (David I). Concerning the surgical procedure, the choice between a mechanical and biological valve graft conduit were on the patient's decision. In patients with pure aortic insufficiency and macroscopically normal aortic leaflets the decision to perform valve sparing aortic root reimplantation was dictated by the surgeon's preference.

**Table 1:** Preoperative patient's characteristics and hemodynamics

Variable	1. MECH-group n = 51	2. BIO-group n = 22	3. REIMPL-group n = 22	All n = 95	*p*-value
Age at surgery (years)					
mean ± SD	73.1 ± 2.7	75.2 ± 3.8	74.1 ± 3.7	73.8 ± 3.2	0.031
range	70–80	70–82	70–83	70–83	
Male, n (%)	29 (56.9)	9 (40.9)	10 (45.5)	48 (51)	n.s.
BSA (m^2^)	1.83 ± 0.21	1.78 ± 0.19	1.84 ± 0.24	1.82 ± 0.21	n.s.
NYHA (mean ± SD)	3.06 ± 0.81	3.0 ± 0.58	2.62 ± 0.74	2.94 ± 0.75	0.007
NYHA ≥ III, n (%)	40 (78.4)	19 (86.4)	15 (68.2)	69 (72.6)	0.031
Redo, n (%)	16 (31.4)	1 (4.5)	0	17 (17.9)	0.001
Endocarditis, n (%)	4 (7.8)	1 (4.5)	0	6 (6.3)	n.s.
Ø ascending aorta (mm)	63 ± 13	62 ± 10	65 ± 13		
*Comorbitities*, n (%)					
AF	12 (23.5)	7 (31.8)	2 (9.1)	21 (22.1)	n.s.
MI	4 (7.8)	3 (18.1)	0	7 (7.3)	n.s.
Ventricular arrhythmias	2 (3.9)	0	0	2 (2.1)	n.s.
Heart failure history	24 (47.1)	8 (36.4)	4 (18.2)	36 (37.9)	n.s.
Systemic hypertension	26 (51)	16 (72.7)	18 (81.2)	60 (63.2)	0.026
Diabetes	5 (9.8)	3 (13.6)	3 (13.6)	11 (11.6)	n.s.
Hyperlipidemia	9 (17.6)	9 (40.9)	7 (31.8)	25 (26.3)	n.s.
Creatinine ≥ 120 μmol/L	9 (17.6)	7 (31.8)	2 (9.1)	17 (17.9)	n.s.
COPD	9 (17.6)	5 (22.7)	2 (9.1)	16 (16.8)	n.s.
Stroke	1 (1.9)	3 (13.6)	2 (9.1)	6 (6.3)	n.s.
TIA/PRIND	2 (3.9)	1 (4.5)	0	4 (3.1)	n.s.
*Hemodynamics*					
LVEF (%)	56.8 ± 11.5	58.6 ± 13.3	60.7 ± 13.8	58.3 ± 12.5	n.s.
LVEDP (mmHG)	17.2 ± 9.4	15.1 ± 6.2	15.7 ± 6.9	16.4 ± 8.2	n.s.
mean PAP (mmHG)	25.6 ± 9.6	26.8 ± 11	21 ± 6	24.8 ± 9.4	n.s.
CI (L×min^-1 ^×m^-2^)	2.7 ± 1.5	2.4 ± 0.5	2.7 ± 0.9	2.6 ± 0.9	n.s.
mean RAP (mmHG)	5.9 ± 3.8	7.8 ± 3.3	6.9 ± 3.1	6.7 ± 3.5	n.s.

BSA, *body surface area*; NYHA, *New York Heart Association*; AF, *Atrial fibrillation*; MI, *previous myocardial infarction*; COPD, *chronic obstructive pulmonary disease*; TIA, *transient ischemic attack*; PRIND, *prolonged reversible ischemic neurological deficit*; LVEF, *left ventricular ejection fraction*; LVEDP, *left ventricular endiastolic pressure*, PAP, *pulmonary artery pressure*; CI, *cardiac index*; RAP, *right atrial pressure*.

We obtained clinical data by retrospective review of the hospital archives and follow-up information was gathered from direct telephone interview of the patient or a close relative, and contact with the referring physician. Infectious, thrombembolic and bleeding complications were recorded as required by the guidelines of the American Association for Thoracic Surgery/Society of Thoracic Surgeons [14]. Furthermore current NYHA-Status and performance status scale (Karnofsky) were requested [15].

Thromboembolism, hemorrhage requiring hospitalisation, aortic valve re-operations or endocarditis were considered to be valve related. Follow-up was complete for 95.2% (79/83) of hospital survivors.

### Surgical technique

Standard cardiopulmonary bypass with a membrane oxygenator and systemic moderate hypothemia (28°C to 32°C) was used unless otherwise indicated. In cases with aortic arch aneurysms either moderate hypothermic circulatory arrest with cold (15°C) antegrade cerebral perfusion or deep hypothermia (16°C to 20°C) with circulatory arrest was utilized [16]. Antegrade cold potassium crystalloid or blood cardioplegic solution was used for myocardial protection. The left ventricle was vented through a transmitral catheter. The decision whether to perform valve sparing aortic root reimplantation or composite replacement of the aortic root was made intraoperatively in patients with pure aortic regurgitation, and depended on the quality of the aortic valve leaflets as well as on the surgeon's decision. In patients with aortic stenosis or mixed aortic valve pathology either a mechanical or a biological composite valve graft was implanted. All patients included in this study receiving a mechanical or biological composite valve graft (CVG) had a button Bentall operation [4]. Biological conduits were manufactured by selecting appropriate size valve prosthesis and suturing a vascular graft to it, yielding a readily usable unit as described by Galla and co-workers [17]. Patients selected for valve sparing aortic root replacement were operated upon the classic David I technique as described previously [18].

The associated operative procedures as well as operative data are summarized in Table 2.

**Table 2:** Intraoperative data

Variable	1. MECH-group n = 51	2. BIO-group n = 22	3. REIMPL-group n = 22	All n = 95	*p*-value
CPB time (min.)	144 ± 60	178 ± 140	159 ± 27	156 ± 82	n.s.
ACC time (min.)	91 ± 31	107 ± 39	123 ± 18	103 ± 33	<0.001
AA-replacement, n (%)	16 (31.4)	15 (68.2)	9 (40.9)	40 (42.1)	0.014
hemiarch, n (%)	15 (93.8)	15 (100)	7 (77.8)	37	0.018
total arch, n (%)	1 (6.2)	0	2 (22.2)	3	n.s.
CA time (min.)	15.5 ± 5.7	17.1 ± 8	17.7 ± 7.7	16.6 ± 7	n.s.
SACP, n (%)	7 (44.4)	15 (100)	6 (66.7)	28 (70)	<0.001
MVS	4 (7.8)	3 (13.6)	0	7	n.s.
CABG, n (%)	11 (21.6)	9 (40.9)	7(31.8)	28 (29.5)	n.s.
Intraoperative death, n (%)	2 (3.9)	0	0	2 (2.1)	n.s.

CPB, *cardio pulmonary bypass*; ACC, *aortic cross clamp*; AA-replacement, *aortic arch replacemen*; SACP, selective antegrade cerebral perfusion, CA, *circulatory arrest*; MVS, *mitral valve surgery*, CABG, *coronary artery bypass grafting*.

### Statistical methods

Continuous variables are expressed as mean ± SD. Categoric data are given as total numbers and relative frequencies; continuous data are given as mean ± SD, except where otherwise stated. Groups were compared by one way ANOVA. Stepwise logistic regression was used for multivariate analysis. The Kaplan-Meier survival estimates were used to analyze long-term survival, and freedom from valve related complications. Statistical differences in Kaplan-Meier survival estimates were determined by using the log-rank test. All data analyses were performed with SPSS 15.0 for Windows (SPSS, Chicago, IL, USA).

## Results

As a result of the surgical policy, there was a consistently increasing caseload of patient receiving either a biological composite graft or a valve sparing aortic root reimplantation over the study period (1988–1992: MECH 12 pts., 1993–1998: MECH 13 pts., BIO 3 pts., REIMPL 2 pts., 1998–2003: MECH 26 pts., BIO 19 pts., REIMPL 20 pts.).

Patient's preoperative data are depicted in Table 1. The patient groups were comparable beside the age, incidence of previous cardiac surgery and arterial hypertension as well as NYHA-status. Preoperative hemodynamics did not show any significant differences between groups. Five patients that were operated on an emergency basis received a mechanical conduit.

Intraoperative variables are shown in Table 2. The REIMPL-group had a significantly longer aortic cross clamp time compared to the MECH-group and BIO-group. The most frequent concomitant surgical procedure was aortic arch replacement (42.1%) and coronary artery bypass grafting (CABG) (29.5%). However, the BIO-group had a significantly higher number of aortic arch replacement performed compared to the MECH-group, furthermore, the percentage of selective antegrade cerebral perfusion (SACP) for cerebral protection was significantly higher in this BIO-group compared to the other patient groups. SACP is routinely performed in our institution since 1999.

### Hospital morbidity and mortality

Early postoperative complications are shown in Table 3. The most frequent complications were postoperative pulmonary insufficiency requiring prolonged ventilation of patients and rethoracotomy for bleeding complications, with no statistical differences between groups. Postoperative pulmonary insufficiency occurred more often in the BIO-group (36.4%) (p = 0.013). 10 patients died within 30 days, in addition to the two intraoperative death the overall hospital mortality was 12.6% with a statistical significant difference between groups (p = 0.004). The causes of death are summarized in Table 4. Stepwise logistic regression revealed a history of preoperative cardiac decompensation (p = 0.026, OR 3.9 (1.1–14.2 95% CI)), rethoracotomy (p = 0.001, OR 12.8 (3.1–52.2 95% CI)) and postoperative pneumonia (p = 0.002, OR 8.1 (1–64 95% CI)) as independent predictors for in hospital mortality.

**Table 3:** Postoperative data of patients surviving the operation

Variable	MECH-group n = 49	BIO-group n = 22	REIMPL-group n = 22	All n = 95	*p*-value
ICU stay (days)					
mean ± SD	3.8 ± 6.2	3.7 ± 4	3.2 ± 4.7	3.6 ± 5.3	n.s.
range	1–37	1–15	1–23	1–37	
artificial ventilation (hours)					
mean ± SD	112 ± 187	64 ± 93	45 ± 87.8	79 ± 137	n.s.
range	24–792	12–360	11–160	11–192	
blood loss (ml)	1128 ± 914	926 ± 882	833 ± 650	999 ± 839	n.s.
red blood cells (units)	2.5 ± 2.8	3.9 ± 3.5	2.9 ± 2	3.2 ± 3	n.s.
fresh frozen plasma (units)	3 ± 2.1	3.4 ± 1.9	2.7 ± 1.86	3.1 ± 1.9	n.s.
rethoracotomy, n (%)	6 (11.8)	5 (22.7)	1 (4.5)	12 (12.9)	n.s.
LCO, n (%)	2 (3.9)	1 (4.5)	0	5	n.s.
MOF, n (%)	3 (5.9)	3 (13.6)	0	6	n.s.
MI, n (%)	0	1 (4.5)	0	1	n.s.
Stroke, n (%)	1 (2)	1 (4.5)	0	3	n.s.
Pneumonia, n (%)	4 (7.8)	0	0	3	n.s.
Sepsis, n (%)	4 (7.8)	1 (4.5)	0	5	n.s
Pulmonary insufficiency, n (%)	7 (13.7)	8 (36.4)	1 (4.5)	16	0.013
Hospital mortality, n (%)	9 (17.6)	3 (13.5)	0	12 (12.6)	0.004

ICU, intensiv care unit; LCO, *low cardiac output*; MOF, *multiorgan failure*; MI, *myocardial infarction*.

**Table 4:** Causes of hospital mortality according to the surgical technique

Cause of hospital mortality	MECH-group n = 51	BIO-group n = 22	REIMPL-group n = 22	All n = 95
Heart failure (intraoperative), n (%)	2 (3.9)			2 (2.1)
Heart failure (postoperative), n (%)	4 (7.8)	1 (4.5)		5 (5.3)
Myocardial infarction, n (%)		1 (4.5)		1 (1.0)
Sepsis, n (%)	2 (3.9)			2 (2.1)
Stroke, n (%)	1 (2)	1 (4.5)		2 (2.1)
Σ hospital mortality, n (%)	9 (17.6)	3 (13.5)	0	12 (12.6)

### Follow-up morbidity and mortality

**Figure 1:**
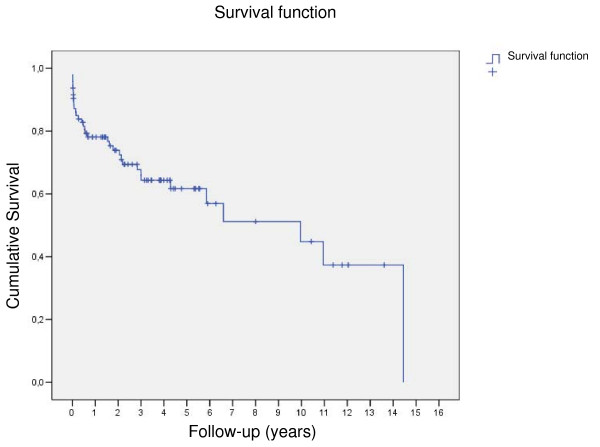
**Survival curve for the entire study population**.

**Figure 2:**
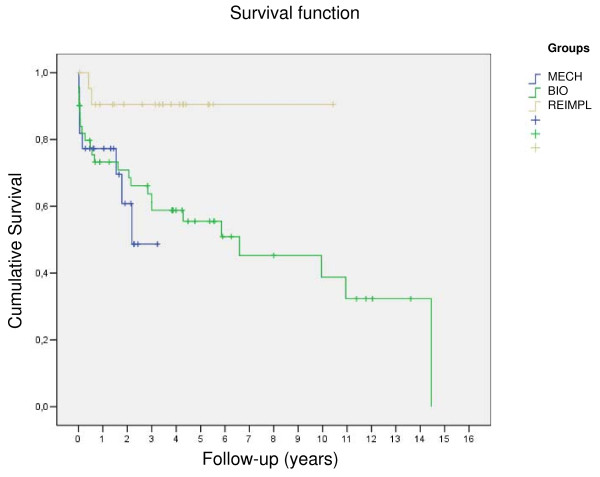
**Survival curves for the sub groups**.

The follow-up was 41 ± 42.3 months (1–173 months) for the entire cohort. The follow-up time showed a significant difference between groups (MECH-group 49 ± 48 months, BIO-group 25 ± 36.9 months, and REIMPL-group 40 ± 28 months, p = 0.028). Follow-up revealed 22 late deaths, 6 patients died of cardiac related reason, 3 of valve related reasons. Thus mean survival for the entire patient population was 8 ± 1 years and showed significant difference between groups as depicted in Figure 1 and Figure 2.

23 hospital survivors had to be readmitted to the hospital, 7 for valve related complications, 2 for cardiac related complications and 14 for other reasons. During follow-up 3 patients had to be re-operated on, all patients had prosthetic valve endocarditis, two of these patients died (freedom from re-operation 96.2%).

**Figure 3:**
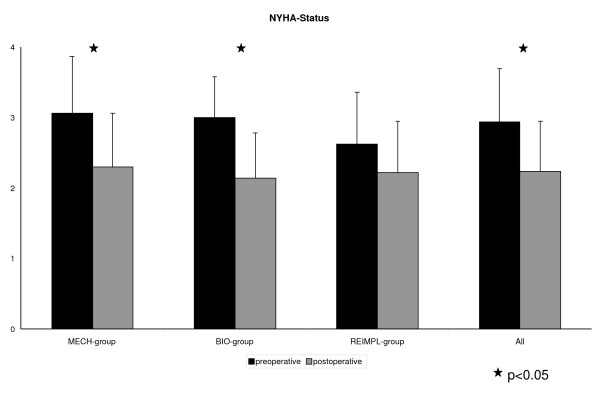
**NYHA-Status of the patients pre- and postoperatively**.

Postoperative NYHA-Status decreased significantly for the entire cohort as well as patients in the MECH-group and BIO-group (Figure 3). The performance status of the entire group (78 ± 7.5% to 81.1 ± 13.2%, p = 0.017) as well for the MECH-group (76.5 ± 7.8% to 82.6 ± 12.5%, p = 0.015) and BIO-group (76.4 ± 8.1% to 81.3 ± 14.1%, p = 0.027) slightly increased.

## Discussion

In this study we demonstrated that different complex surgical techniques for ARR can be performed with good results in septuagenarians. Our data indicate that valve sparing aortic root reimplantation is an excellent alternative to composite conduits with bioprostheses or mechanical prostheses for septuagenarians with favourable short and long-term outcome.

Composite valve replacement has become a generally accepted surgical technique for the treatment of aortic root pathology with acceptable perioperative mortality rates and good long-term results [6,8,9,11,12,18-21]. The mortality rate for ARR with composite valve grafts in recent publications varies between 0.7% and 11% with a survival rate from 76% to 91.8% at 5 years and 62% to 76% at 10 years. Furthermore, Urbanski and associates showed in a case matched study with 200 patients, that ARR can be performed with a similar operative risk, valve related morbidity and late mortality as isolated aortic valve replacement [21]. However, recent published series showed that valve sparing aortic root replacement techniques are a valuable option for patients with macroscopically normal leaflets [3,10]. The hospital mortalities in these studies vary around 3–5%. The functional results showed freedom from re-operation in 94 – 99% of different sub cohorts.

In the vast majority of these studies the patients were younger. Due to the limited data from the literature there is still an ongoing debate whether ARR should be performed in elderly patients >70 years due to an anticipated higher mortality rate for ARR compared to isolated aortic valve replacement. Only sparse data are available from the literature dealing with the problem of ARR in septuagenarians [8]. Ehrlich and co-workers described a hospital mortality of 8.3% (7 of 84). This is comparable to the 5%–11.4% hospital mortality after aortic valve replacement in the same age group [22-24].

In our study, two patients died intra-operatively, and another 10 within the first 30 day or during hospital stay (12/95 pts., 12.6%). Our institutional mortality for this age group ranges from 8% for cardiac surgery up to 29% in patients undergoing surgery for acute type A aortic dissection [25,26]. In other studies we revealed age and co-morbidities as predictors for morbidity and mortality [27,28]. We strongly believe that the mortality rate is influenced by the amount of co-morbidities rather than the surgical technique.

The standard approach to ARR is the use of composite grafts with a mechanical prosthesis [29]. Composite conduits with stented bioprosthesis have shown reasonable results [9,17]. Etz and associates recently published their experience with 206 patients receiving custom made composite conduits with a stented bioprosthesis (mean age 53 years) with a hospital mortality rate of 2.9% and a 10 year survival rate of 89% [9]. Only a limited number of reports compared mechanical and biological ARR [6,8]. Ehrlich and co-workers showed that overall probability of survival was similar for patients with composite grafts incorporating a mechanical or biological valve (mean age 73.9 years; n = 84) with a 5 year survival of approximately 60% in both groups. Interestingly in this study survival was similar to that of an age-matched population. Byrne et al. compared biological and mechanical root replacement in a younger patient cohort with a median age of 53 years respectively 54 years and could neither demonstrate a difference in hospital mortality (1.5% vs. 2.4%) nor in the 5 year survival rate (92.4% vs. 88.2%). However, in the biological root replacement group either aortic homografts or stentless aortic valves were implanted.

In the present study, mean survival was 7 ± 1 years for the entire cohort, with best results for patients in the REIMPL-group (5-year survival rate 80%). The impact of pure aortic insuffiency on results in this subgroup in controversially discussed. A substantial number of patients receiving a mechanical or biological composite graft had either mixed AS/AI or AS only; in contrast patients scheduled for aortic root reimplantation had pure AI only. The impact of different physiological subgroups (aortic regurgitation vs. aortic stenosis vs. stenosis/regurgitation) on early and late outcome after aortic valve surgery is still discussed controversially. Scott and associates analysed 1,479 isolated aortic valve replacement procedures and showed substantial differences in operative mortality rates among physiological subgroups [30]. In contrast Nowicki and colleagues reported on 5793 patients undergoing aortic valve surgery and found no impact of valve diagnosis on in-hospital mortality [31]. However, in both studies no long-term results have been analysed.

For surgeons not familiar with valve sparing aortic root procedures ARR with composite conduits is still a valuable option. Whereas in this patient composite graft replacement with stented bioprosthesis is omitting lifelong oral anticoagulation und consequent avoidance of bleeding complications [32].

### Limitations

Patient's demographic and intraoperative data differed significantly between groups in terms of number of previous cardiac operations, preoperative NYHA functional class, and incidence of aortic arch replacement, all factors that may reflect a selection bias for a certain operative technique. However, the multivariate analysis revealed none of these factors as a predictor for hospital mortality. Since this is a nonrandomised retrospective study covering the experience of 15 years, factors influencing the outcome may have been missed and not analysed thus could falsify the result of the multivariate analysis. However, current factors that already proofed to have a potential influence as predictors for hospital mortality and long-term mortality have been proven.

## Conclusion

The small number of patients in each group makes it difficult to draw definite conclusion about the impact of different surgical techniques for ARR on early and late outcome.

However, our data indicate that valve sparing aortic root Reimplantation is safe and effective in septuagenarians, and is associated with low early and late morbidity and mortality.

## Competing interests

The authors declare that they have no competing interests.

## Authors' contributions

NK and RL designed the study and wrote the manuscript, MS and SP were responsible for data acquisition, interpretation and statistical analysis, AH and CH revised the manuscript for important intellectual content and have given the final approval of the version to be published.
